# Dimensions of Emotional Intelligence and Online Gaming Addiction in Adolescence: The Indirect Effects of Two Facets of Perceived Stress

**DOI:** 10.3389/fpsyg.2017.01206

**Published:** 2017-07-13

**Authors:** Dexin Che, Jianping Hu, Shuangju Zhen, Chengfu Yu, Bin Li, Xi Chang, Wei Zhang

**Affiliations:** ^1^Laboratory for Behavioral and Regional Finance, Guangdong University of Finance Guangzhou, China; ^2^School of Psychology and Center for Studies of Psychological Application, South China Normal University Guangzhou, China; ^3^School of Education and Center for Mind and Brain Science, Guangzhou University Guangzhou, China

**Keywords:** emotional intelligence, perceived helplessness, perceived self-efficacy, online gaming addiction, adolescence

## Abstract

This study tested a parallel two-mediator model in which the relationship between dimensions of emotional intelligence and online gaming addiction are mediated by perceived helplessness and perceived self-efficacy, respectively. The sample included 931 male adolescents (mean age = 16.18 years, *SD* = 0.95) from southern China. Data on emotional intelligence (four dimensions, including self-management of emotion, social skills, empathy and utilization of emotions), perceived stress (two facets, including perceived self-efficacy and perceived helplessness) and online gaming addiction were collected, and bootstrap methods were used to test this parallel two-mediator model. Our findings revealed that perceived self-efficacy mediated the relationship between three dimensions of emotional intelligence (i.e., self-management, social skills, and empathy) and online gaming addiction, and perceived helplessness mediated the relationship between two dimensions of emotional intelligence (i.e., self-management and emotion utilization) and online gaming addiction. These findings underscore the importance of separating the four dimensions of emotional intelligence and two facets of perceived stress to understand the complex relationship between these factors and online gaming addiction.

## Introduction

Online gaming has become popular in the daily life of adolescents. A series of studies have shown that adolescents with excessive online gaming may display several classic signs of addiction, including being preoccupied by online games, causing various family and relationship problems, and experiencing mood modification (Griffiths et al., 2015). This has motivated researchers to devote effort to understand the psychological mechanisms of online gaming addiction. Part of these efforts have been devoted to identify both protective and risk factors for online gaming addiction in adolescence (Koo and Kwon, 2014).

Dual Systems Model of adolescent problem behavior (Somerville et al., 2010; Steinberg, 2010) proposed that, increasing problematic behaviors during adolescence are the product of the more mature limbic (socio-emotional) system winning over the prefrontal (cognitive control) system. The current study introduced the variable of trait emotional intelligence, which describes “a constellation of behavioral dispositions and self-perceptions concerning one’s ability to recognize, process, and utilize emotion-laden information” (Petrides and Furnham, 2001). Trait emotional intelligence reflects the coordination of these two systems (Barbey et al., 2012; Panno, 2016; Semper et al., 2016). It is a multidimensional construct. According to Chan (2003) and Li et al. (2009), trait emotional intelligence can be separated into four dimensions, namely, self-management of emotions (i.e., self-awareness, understanding and regulation of one’s own emotions), social skills (i.e., explicit actions that individuals take to respond to social tasks), empathy (i.e., the awareness and management of others’ emotions) and utilization of emotions (i.e., an individual’s use of emotions). Numerous studies suggest that, emotional intelligence was positively associated with improved psychological health (Fernández-Abascal and Martín-Díaz, 2015), as well as negatively associated with problem behaviors (Siu, 2009; Gugliandolo et al., 2015). However, so far, only a few studies have investigated the associations between emotional intelligence and internet addiction in adolescence (Parker et al., 2008; van Deursen et al., 2015). In these limited numbers of empirical studies, some studies have reported a negative association between some specific dimensions of emotion intelligence (e.g., self-management of emotions, social skills and empathy) and internet addiction (Engelberg and Sjöberg, 2004; Oktan, 2011; Melchers et al., 2015), while some other studies have failed to find significant relationship between general emotional intelligence and internet addiction/online gaming addiction (van Deursen et al., 2015). One potential reason for these discrepancies is that whether investigators take emotional intelligence as a multi-dimensional construct. Without controlling for the influence of other dimensions of emotional intelligence or taking emotional intelligence as a unidimensional construct may exacerbate or mask the association between emotional intelligence and internet addiction/online gaming addiction. Thus, more research simultaneously exploring the role of these four dimensions of emotional intelligence with relation to online gaming addiction is needed, and our study aims to address this gap in the literature.

Another gap is the lack of research on the mediating process that may explain the relationship between emotional intelligence and online gaming addiction. One potential mechanism is through perceived stress. Perceived stress is defined as the degree to which situations in one’s life are considered stressful (Cohen et al., 1983). Perceived stress includes two facets, that is, perceived self-efficacy and perceived helplessness (Lee, 2012). Perceived self-efficacy refers to people’s confidence in their ability to manage stressor, whereas perceived helplessness indicates people’s feeling of unable to cope or exert control. To our knowledge, a limited number of studies have shown that perceived stress emerged as a significant mediator of emotional intelligence on psychological well-being (Ruiz-Aranda et al., 2014; Urquijo et al., 2015). However, research has yet to examine the possible mediating role of perceived stress in the link between emotional intelligence and online gaming addiction.

Some indirect evidence has implied that perceived stress mediates the relationship between emotional intelligence and online gaming addiction in adolescence. Core characteristics of emotional intelligence may help understand the relationship between emotional intelligence and perceived stress. The self-regulated functioning inherent in emotional intelligence may facilitate recovery from psychological distress and minimizes susceptibility to the deleterious effects of stress, thus leading to more perceived self-efficacy and less perceived helplessness (Perera and DiGiacomo, 2013, 2015). Segrin et al. (2007) pointed out that, people with good social skills (a key component of emotional intelligence) may be more likely to develop effective coping strategies based on their past success, contributing to greater feelings of self-efficacy. Moreover, some empirical evidences suggest that higher level of emotional intelligence is associated with lower level of perceived stress (Gohm et al., 2005; Mikolajczak and Luminet, 2008; Ruiz-Aranda et al., 2014; Urquijo et al., 2015). Mikolajczak and Luminet (2008) found that, individuals with high trait emotional intelligence exhibited greater self-efficacy to cope. In the study of Chan (2005), adolescents higher on self-management of emotions and the utilization of emotions engaged more in avoidant coping, while adolescents higher on empathy and social skills engaged more in social-interaction coping. It suggested that different dimensions of emotional intelligence might be responsible for different use of social coping strategies, resulting in different perceptions of self-efficacy and helplessness.

On the other hand, recent studies have shown that perceived stress may impact one’s addictive behavior (Velezmoro et al., 2010; Kardefelt-Winther, 2014; Park, 2014; Samaha and Hawi, 2016). For example, several studies have found a positive correlation between perceived stress and internet addiction (and/or online gaming addiction) (Kardefelt-Winther, 2014; Park, 2014). Perceived self-efficacy was found negatively associated with internet addiction (İskender and Akin, 2010). However, in the study of Velezmoro et al. (2010), perceived helplessness, not perceived self-efficacy was found to be related to internet addiction, suggesting the different roles of perceived helplessness and perceived self-efficacy in internet addiction and/or online gaming addiction. Taken together, emotional intelligence may be indirectly associated with online gaming addiction via perceived self-efficacy and perceived helplessness operating in parallel. However, to date, no known studies have yet directly examined the two-mediator (perceived helplessness and perceived self-efficacy) model for explaining the relationship between emotional intelligence and online gaming addiction.

The current study extends previous research by examining the associations between four dimensions of emotional intelligence (i.e., self-management of emotions, the utilization of emotions, empathy and social skills), two facets of perceived stress (i.e., perceived self-efficacy and perceived helplessness) and online gaming addiction in male adolescents. Only male adolescents were recruited since male adolescents spent more time playing online games, were more addicted to online games, had a much higher prevalence of online gaming addiction, and exhibited different mechanisms for online gaming addiction (Ko et al., 2005; Li and Wang, 2013; Ha and Hwang, 2014). This study has two specific goals. Firstly, to determine which dimensions of emotional intelligence have significant and unique associations with online gaming addiction; secondly, to examine whether perceived self-efficacy and perceived helplessness would mediate in parallel the associations between four dimensions of emotional intelligence and online gaming addiction.

In summary, three hypotheses and one research question were formulated. First, following Engelberg and Sjöberg (2004), Oktan (2011), and Melchers et al. (2015), we assumed that social skills, self-management of emotions and empathy would negatively predict online gaming addiction in adolescence. Second, following Gohm et al. (2005) and Velezmoro et al. (2010), we predicted that emotional intelligence would decrease perceived helplessness, which in turn contributes to more online gaming addiction in adolescence. Third, following Mikolajczak and Luminet (2008) and İskender and Akin (2010), we predicted that emotional intelligence would increase perceived self-efficacy, which in turn contributes to less online gaming addiction in adolescence. In addition, we explored the associations between four dimensions of emotional intelligence and the two facets of perceived stress.

## Materials and Methods

### Sample and Procedure

Participants were recruited from two middle schools in southern China. The original sample consisted of 1022 male adolescents from Grade 10 to Grade 12. Of these, 91 (8.9%) were excluded because they had no experience playing online games, resulting in the current sample of 931 male adolescents. The mean age of this sample was 16.18 years (standard error, *SD*, 0.95), ranging from 13 to 19 years.

Written informed consents were obtained from the school, all participants and their parents. Participants in this study were voluntary and anonymous. They were given approximately 30 min to complete the questionnaires in their classrooms. All materials and procedures were approved by South China Normal University Human Investigation Committee.

### Measures

#### Perceived Stress

Adolescents were assessed by the Chinese version of Perceived Stress Scale (PSS; Chu and Kao, 2005). Developed from the PSS (Cohen et al., 1983), the Chinese version of the PSS demonstrated reliability and validity (Chu and Kao, 2005; Leung et al., 2010). It consists of 14 items, which cover two facets: perceived helplessness and perceived self-efficacy. Adolescents were asked to report how often they experienced each symptom in the past month on a 5-point scale ranging from 1 (never) to 5 (very often). The mean of each facet was calculated, with higher mean representing higher level of perceived helplessness and perceived self-efficacy, respectively. The Cronbach’s alpha coefficients of perceived helplessness and perceived self-efficacy in this study were 0.76 and 0.77, respectively.

#### Emotional Intelligence

Adolescents were assessed by the Chinese Emotional Intelligence Scale (Li et al., 2009). Derived from the 33-item long form of the Emotional Intelligence Scale (Schutte et al., 1998; Chan, 2003), the short form of the Chinese Emotional Intelligence Scale demonstrated reliability and validity (Li et al., 2009). It consists of 19 items which cover four dimensions: self-management of emotions (four items), empathy (four items), utilization of emotions (six items), and social skills (five items). Adolescents were asked to rate each item for their agreement using a 5-point scale ranging from 1 (strongly disagree) to 5 (strongly agree). The mean of each dimension was calculated, with higher mean representing higher level of emotional intelligence. The Cronbach’s alpha coefficients were 0.75, 0.75, 0.79, and 0.73 for these four dimensions of emotional intelligence in this study.

#### Online Gaming Addiction

The online gaming addiction scale was modified from the Revised Chinese Internet Addiction Scale (CIAS; Chen et al., 2003) to measure the degree of online gaming addiction tendency in participants. The scale has 26 items and consists of two subscales: Core Symptoms (14 items) and Related Problems (12 items). The former includes three dimensions: compulsive use, withdrawal, and tolerance; the latter includes two dimensions: interpersonal and health-related, and time management problems. For each item, participants indicated how true each statement was for themselves on a 4-point scale ranging from 1 (almost always untrue of you) to 4 (almost always true of you). The mean was taken with a higher mean representing a higher level of online gaming addiction. The Cronbach’s alpha coefficients of Core Symptoms and Related Problems in this study were 0.91 and 0.88, respectively.

### Data Analysis

Missing data (less than 1%) were handled with mean substitution. We first conducted descriptive analyses using SPSS 22. Descriptive statistics and bivariate correlations for the major variables were presented. Second, SPSS MEDIATE macro was used in the analysis of multiple indirect effects (Hayes et al., 2011). All continuous variables were standardized. Bootstrapping method (10,000 bootstrap resamples) was used to test the indirect effect, which is an appropriate test of indirect effect and does not assume the normal distribution of scores for given variables. An indirect path is statistically significant if the associated 95% confidence interval (CI; bias corrected) does not include zero.

## Results

### Preliminary Analyses

Means and standard deviations of the major variables along with their correlations are presented in **Table 1**. Three dimensions of emotional intelligence (self-management of emotions, social skills, and empathy) and perceived self-efficacy were negatively associated with core symptoms and related problems of online gaming addiction. Perceived helplessness was positively correlated with core symptoms and related problems of online gaming addiction.

**Table 1:** Descriptive statistics and intercorrelations for the major variables.

Variables	1	2	3	4	5	6	7	8
1. Self-management	–							
2. Social skills	0.519ˆ***	–						
3. Empathy	0.519ˆ***	0.535ˆ***	–					
4. Emotion utilization	0.457ˆ***	0.576ˆ***	0.519ˆ***	–				
5. Perceived self-efficacy	0.368ˆ***	0.326ˆ***	0.303ˆ***	0.270ˆ***	–			
6. Perceived helplessness	–0.108ˆ**	0.014	–0.001	0.093ˆ**	–0.177ˆ***	–		
7. Core symptoms	–0.232ˆ***	–0.136ˆ***	–0.097ˆ**	–0.058	–0.199ˆ***	0.269ˆ***	–	
8. Negative outcomes	–0.189ˆ***	–0.106ˆ**	–0.092ˆ**	–0.063	–0.178ˆ***	0.248ˆ***	0.827ˆ***	–
*M*	3.665	3.754	3.597	3.758	2.883	2.555	1.994	2.024
*SD*	0.757	0.683	0.747	0.711	0.662	0.650	0.586	0.574

*N = 931; ^∗∗^*p* < 0.01, ^∗∗∗^*p* < 0.001.*

Four dimensions of emotional intelligence were positively associated with perceived self-efficacy. Self-management of emotions and utilization of emotions were negatively and positively associated with perceived helplessness, respectively.

### Regression Analyses of Multiple Indirect Effect

**FIGURE 1:**
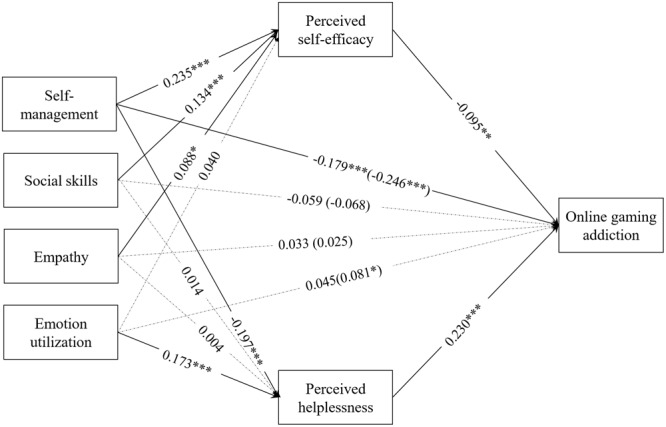
Effects of emotional intelligence on online gaming addiction via perceived self-efficacy and perceived helplessness operating in parallel. ^∗^*p* < 0.05, ^∗∗^*p* < 0.01, ^∗∗∗^*p* < 0.001.

SPSS MEDIATE macro was used in the analysis of multiple indirect effects (Hayes et al., 2011). Results of these analyses are presented in **Tables 2, 3** and summarized in **Figure 1**.

**Table 2:** Effects of four dimensions of emotional intelligence and two facets of perceived stress on online gaming addiction.

	Criterion: online gaming addiction			Criterion: online gaming addiction		
Predictors	*b*	*SE*	*t*	*b*	*SE*	*t*
Self-management	–0.246ˆ***	0.040	–6.168	–0.179ˆ***	0.040	–4.508
Social skills	–0.068	0.043	–1.588	–0.059	0.042	–1.406
Empathy	0.025	0.041	0.609	0.033	0.040	0.816
Emotion utilization	0.081ˆ*	0.041	1.961	0.045	0.040	1.120
Perceived self-efficacy				–0.095ˆ**	0.034	–2.769
Perceived helplessness				0.230ˆ***	0.032	7.200

*N = 931; ^∗^*p* < 0.05, ^∗∗^*p* < 0.01, ^∗∗∗^*p* < 0.001.*

**Table 3:** Effects of four dimensions of emotional intelligence on perceived self-efficacy and perceived helplessness, respectively.

	Criterion: perceived self-efficacy			Criterion: perceived helplessness		
Predictors	*b*	*SE*	*t*	*b*	*SE*	*t*
Self-management	0.235^∗∗∗^	0.038	6.251	–0.197^∗∗∗^	0.040	–4.869
Social skills	0.134^∗∗∗^	0.040	3.324	0.014	0.043	0.323
Empathy	0.088^∗^	0.039	2.275	0.004	0.042	0.095
Emotion utilization	0.040	0.039	1.031	0.173^∗∗∗^	0.042	4.134

*N = 931; ^∗^*p* < 0.05, ^∗∗∗^*p* < 0.001.*

As seen in **Table 2**, without controlling for the influence of the mediators, only two dimensions of emotional intelligence had significant effect on online gaming addiction: self-management of emotion (*b* = -0.246, *p* < 0.001) and emotion utilization (*b* = 0.081, *p* < 0.05). Hypothesis 1 was partially supported.

As seen in **Table 3** and **Figure 1**, three dimensions of emotional intelligence had significant direct paths to the mediator of perceived self-efficacy: self-management of emotion (*b* = 0.235, *p* < 0.001), social skills (*b* = 0.134, *p* < 0.001) and empathy (*b* = 0.088, *p* < 0.05). Two dimensions of emotional intelligence had significant direct paths to the mediator of perceived helplessness: self-management of emotion (*b* = -0.197, *p* < 0.001) and emotion utilization (*b* = 0.173, *p* < 0.001). As shown in **Table 2**, two mediators had significant direct paths to online gaming addiction: perceived self-efficacy (*b* = -0.095, *p* < 0.01) and perceived helplessness (*b* = 0.230, *p* < 0.001).

Further, as seen in **Table 4**, using 10,000 bootstrap resampling, the lower and upper values of the 95% confidence interval for the indirect effects of self-management, social skills, and empathy through perceived self-efficacy (*b* = -0.022, *CI* = [-0.045, -0.005]; *b* = -0.013, *CI* = [-0.030, -0.003]; *b* = -0.008, *CI* = [-0.023, -0.001], respectively), and the indirect effects of self-management and emotion utilization through perceived helplessness (*b* = -0.045, *CI* = [-0.073, -0.025]; *b* = 0.040, *CI* = [0.018, 0.068], respectively), did not include zero, demonstrating that these indirect effects were significant. Therefore, Hypothesis 2 and Hypothesis 3 were partially supported.

**Table 4:** Results of indirect effect through perceived self-efficacy and perceived helplessness, respectively.

	Mediator: perceived self-efficacy				Mediator: perceived helplessness			
Predictors	*b*	*SE*	*LLCI*	*ULCI*	*b*	*SE*	*LLCI*	*ULCI*
Self-management	–0.022^a^	0.010	–0.045	–0.005	–0.045^a^	0.012	–0.073	–0.025
Social skills	–0.013^a^	0.007	–0.030	–0.003	0.003	0.012	–0.020	0.027
Empathy	–0.008^a^	0.005	–0.023	–0.001	0.001	0.010	–0.019	0.022
Emotion utilization	–0.004	0.004	–0.015	0.002	0.040^a^	0.013	0.018	0.068

*N = 931.^a^ Empirical 95% confidence does not overlap with zero.*

## Discussion

In the current study, we investigated the associations between four dimensions of emotional intelligence (i.e., self-management of emotions, emotion utilization, empathy and social skills), two facets of perceived stress (i.e., perceived self-efficacy and perceived helplessness) and online gaming addiction in adolescence. When comparing our results with the literature, there are some consistent and different, even surprising findings. This study contributes to a growing body of literature in at least five ways.

First, we found that different dimensions of emotional intelligence played different roles in online gaming addiction. Specifically, self-management of emotions and emotion utilization were negatively and positively related to online gaming addiction, respectively. Using the general or the specific component of trait emotional intelligence to examine effects on psychological well-being or problem behavior may mask or exacerbate some relationships. While simultaneously including four dimensions of emotional intelligence in the regression analyses, the distinct and unique effects of each dimension can be revealed with controlling for other dimensions. Meanwhile, it is worth noting that in correlation analysis, three dimensions of emotional intelligence were negatively associated with online gaming addiction. However, the associations between emotion utilization and online gaming addiction became significantly positive when variances in other three dimensions of emotional intelligence were taken into account in the regression analysis. This indicated that the remaining part of emotion utilization variance was positively associated with online gaming addiction. This surprising result is consistent with a recent evidence showing that emotional intelligence has a deleterious influence on non-clinical depressive symptomatology in high school students (Lombas et al., 2014). A non-clinical sample is also investigated in the current study. It might be the case that emotion utilization plays a specific role on the early stage of online gaming addiction. In addition, emotion utilization refers to the use of the energy of emotion arousal (Izard et al., 2011). Utilizing emotions requires utilizing feelings to assist with problem solving, which requires the balance between socio-emotional system and cognitive control system. However, according to the Dual Systems Model (Steinberg et al., 2008; Somerville et al., 2010), the immature cognitive control system cannot restrain the heightened reactivity of the social-emotional system during adolescence. High emotional arousal has the potential to undermine the immature cognitive system (Botdorf et al., 2016). One is tempted to speculate that, after controlling for the effects of variance in three dimensions of emotional intelligence, adolescents with immature cognitive control system were unable to override the negative impact of positive affective feelings with online games, resulting in higher tendency of online gaming addiction (Hu et al., 2017).

Second, perceived helplessness was found to mediate the relationship between two dimensions of emotional intelligence (i.e., self-management and emotion utilization) and online gaming addiction. At the first stage of the mediation analysis, self-management of emotions was associated with less perceived helplessness, whereas emotion utilization was associated with greater level of perceived helplessness. The positive relationship between emotion utilization and perceived helplessness is somewhat surprising; however, it may help us understand the positive association between emotion utilization and online gaming addiction. Emotion utilization is not always beneficial. Efforts directed toward self-management, social skills, and emotion understanding may facilitate emotion utilization (Izard et al., 2008). After controlling for the effects of self-management, social skills and empathy, the maladaptive function of emotion utilization may be revealed. In addition, maladaptive affective-cognitive information processing degrades emotion utilization (Izard et al., 2008; Izard et al., 2011). Mai et al. (2012) pointed out that, internet addiction-related maladaptive cognitions (i.e., social comfort, distraction and self-realization) play an important role in the onset and development of internet addiction among Chinese adolescents. Given the “strength” of socio-emotional system exceeds that of cognitive control system, these maladaptive emotion-related cognitive content may become overwhelming, resulting in the perception of lacking control over the reality and perceived helplessness. At the second stage of mediation analysis, perceived helplessness was positively associated with online gaming addiction. Consistent with the current results, perceived helplessness has been identified as the risk factor for internet addiction. For example, individuals who are unable to cope actively with stressful situation or who have high level of negative affectivity are more likely to have internet addiction tendency (Kim et al., 2006; Kuss and Griffiths, 2012; Odacı and Celik, 2016). Only few studies have investigated the associations between emotion utilization, perceived helplessness and online gaming addiction, so these results should not be overstated and more research is needed to replicate these findings.

Third, we found that perceived self-efficacy mediated the relationship between three dimensions of emotional intelligence (i.e., self-management, social skills, and empathy) and online gaming addiction. At the first stage of the mediation analysis, self-management, social skills, and empathy were associated with greater level of perceived self-efficacy. It would appear those who can regulate their own emotions, have adequate social skills and have high levels of emotional understanding, consider themselves able to process the stress, resulting in higher perceived self-efficacy (Davis and Humphrey, 2012).

Fourth, we identified different antecedents of perceived self-efficacy and perceived helplessness. Perceived self-efficacy positively related to self-management, social skills and empathy, whereas perceived helplessness negatively and positively related to self-management and emotion utilization, respectively. Different antecedents of perceived self-efficacy and perceived helplessness support the two-factor structure of the perceived stress, highlighting the importance of recognizing and incorporating the distinction between perceived self-efficacy and perceived helplessness.

Fifth, this study provides evidence that significant indirect effects do not necessarily require a significant total effect. In this study, social skills and empathy appear to exert effects on online gaming addiction through two pathways that work in opposite directions, by increasing perceived self-efficacy, which in turn reduces the tendency of online gaming addiction, while simultaneously increasing perceived helplessness (non-significant), which increases the tendency of online gaming addiction. These two indirect effects tend to offset each other, resulting in non-significant total effects (Hayes, 2009). Without hunting for indirect effect on the non-significant total effects would lead us to miss these important and interesting mechanisms by which social skills and empathy exert effects on online gaming addiction.

We acknowledge several limitations in this study. First, its cross-sectional and correlational design limits the ability to draw any causal inferences. Testing our parallel two-mediator model using longitudinal design may provide additional insights into relationships between these variables. Second, all variables were collected using self-report measure and may be susceptible to social desirability and common method variance problems. Future studies may use multi-method, multi-informant approaches to lower the subjectivity. Third, the current study did not assess participants’ past or current psychiatric symptoms associated with online gaming addiction. Future studies may collect data of psychiatric symptoms, such as depression and social phobia, to control the effect of these psychiatric disorders on online gaming addiction (Andreassen et al., 2016). Fourth, the sample in this study is made up exclusively of male adolescents, thus the current findings may not be generalized to female adolescents. Since there are gender differences in emotional intelligence (Petrides and Furnham, 2006) and the prevalence of internet addiction (Cao and Su, 2007), future research should extend the study to include female adolescents and analyze the existence of possible different patterns regarding how four dimensions of emotional intelligence and two facets of perceived stress relate to online gaming addiction.

Despite its limitations, the current study provides insight into potential pathways toward four dimensions of emotional intelligence and online gaming addiction tendency in a sample of Chinese male adolescents. Specifically, the relationships between three dimensions of emotional intelligence (i.e., self-management, social skills and empathy) and online gaming addiction tendency were mediated by perceived self-efficacy, and the relationships between two dimensions of emotional intelligence (i.e., self-management and emotion utilization) and online gaming addiction tendency were mediated by perceived helplessness. These findings underscore the importance of separating the four dimensions of emotional intelligence and two facets of perceived stress to understand the complex relationship between these variables and online gaming addiction, and provide evidence for the existence of indirect effects on the non-significant total effects.

## Author Contributions

Conceived and designed the research: DC and JH. Performed the research: JH, SZ, and CY. Analyzed the data: DC, JH, SZ, CY, and XC. Contributed to the writing of the manuscript: DC, JH, SZ, CY, BL, XC, and WZ.

## Conflict of Interest Statement

The authors declare that the research was conducted in the absence of any commercial or financial relationships that could be construed as a potential conflict of interest.
